# Analysis of Gut Microbiota Signature and Microbe-Disease Progression Associations in Locally Advanced Non-Small Cell Lung Cancer Patients Treated With Concurrent Chemoradiotherapy

**DOI:** 10.3389/fcimb.2022.892401

**Published:** 2022-06-01

**Authors:** Yu Xi, FangJie Liu, Bo Qiu, Ying Li, XinQiang Xie, JinYu Guo, Lei Wu, TingTing Liang, DaQuan Wang, Juan Wang, Moutong Chen, Liang Xue, Yu Ding, Jumei Zhang, QingPing Wu, Hui Liu

**Affiliations:** ^1^ School of Biology and Biological Engineering, South China University of Technology, Guangzhou, China; ^2^ Guangdong Provincial Key Laboratory of Microbial Safety and Health, State Key Laboratory of Applied Microbiology Southern China, Institute of Microbiology, Guangdong Academy of Sciences, Guangzhou, China; ^3^ Department of Radiation Oncology, State Key Laboratory of Oncology in South China, Collaborative Innovation Center for Cancer Medicine, Sun Yat−sen University Cancer Center, Guangzhou, China

**Keywords:** non-small cell lung cancer, gut microbiota, chemoradiotherapy, disease progression, metagenomes, antibiotic resistance genes

## Abstract

**Purpose:**

To evaluate the association of gut microbiome signature and disease progression in locally advanced non-small cell lung cancer (LA-NSCLC) patients treated with concurrent chemoradiotherapy (CCRT) by fecal metagenome analysis.

**Methods:**

Metagenome-wide association studies on baseline fecal samples from 18 LA-NSCLC patients before CCRT and 13 controls from healthy first-degree relatives were performed. Among the 18 LA-NSCLC patients, six patients were defined as the long progression-free survival (long-PFS) group (PFS≥11 months) while another 12 were in the short-PFS group (PFS<11 months). Alpha diversity, taxonomic composition, and Kyoto Encyclopedia of Genes and Genomes (KEGG) functional pathways were compared between groups.

**Results:**

The Firmicutes/Bacteroidetes value of long-PFS group was higher than those of short-PFS (p=0.073) and healthy individual groups (p=0.009). Meanwhile, long-PFS group had significantly higher diversities in Fungi, Archaea, and Viruses than short-PFS group. The KEGG pathways overrepresented in short-PFS group included fructose and mannose metabolism (p=0.028), streptomycin biosynthesis (p=0.028), acarbose and validamycin biosynthesis (p=0.013), ribosome biogenesis in eukaryotes (p=0.035), biosynthesis of vancomycin group antibiotics (p=0.004), apoptosis-fly (p=0.044), and tetracycline biosynthesis (p=0.044), while those overrepresented in long-PFS group included fatty acid biosynthesis (p=0.035), fatty acid metabolism (p=0.008), vancomycin resistance (p=0.008), longevity regulating pathway-worm (p=0.028), type II diabetes mellitus (p=0.004), and viral carcinogenesis (p=0.003). Further analysis of antibiotic resistome demonstrated that the short-PFS group had a trend with more antibiotic resistance genes than healthy control (p=0.070) and long-PFS groups (p=0.218). The vancomycin resistance sequences were significantly enriched in the long-PFS group compared to the short-PFS group (p=0.006).

**Conclusions:**

The baseline gut microbiome composition and functionality might be associated with PFS in LA-NSCLC treated with CCRT. The outcome of CCRT might be modulated through bacterial metabolic pathways. The antibiotic resistance genes might play a role in disease progression and provide potential information on the relationship between the use of antibiotics and treatment efficacy of CCRT in LA-NSCLC.

## Introduction

Concurrent chemoradiotherapy (CCRT) is regarded as the standard treatment of locally advanced non-small cell lung cancer (LA-NSCLC). However, distant metastases and locoregional recurrences remain major causes of mortality even with the combination of immunotherapy. Recent studies indicate that gut microbial dysbiosis may be an important environmental factor in this subgroup of patients (Liu et al., 2019). LA-NSCLC has traditionally been known to be a complicated disease induced by interactions between the host and the environment (Khan et al., 2021). Microbes, along with other environmental risk factors, play a vital role in maintaining microecological homeostasis and modulating host immunological responses to multiple therapies.

Clinical research has discovered that individuals with gastrointestinal disorders were more prone to develop chronic respiratory diseases (Wang et al., 2013; Stokholm et al., 2018). These substantial findings suggest the existence of a microbial interaction network that alters host vulnerability to pathogenic factors. The gut microbiota has been correlated to chronic obstructive pulmonary disease, asthma, and exacerbating acute lung damage (Molyneaux et al., 2013; Marri et al., 2013; Prakash et al., 2015). An investigation has also revealed that specific microbial metabolites exerted modulatory effects between gut and lung tissue *via* circulation (Perrone et al., 2012). For instance, there was a substantial decline in microbial metabolites such as short-chain fatty acids (SCFA) in asthma patients’ stools compared with healthy controls (Ivashkin et al., 2019). In addition, gut microbes’ fragments were found to be taken up and phagocytosed by macrophages which could migrate into lung *via* circulation to regulate immune response (Bingula et al., 2017).

Lung cancer patients displayed a significant shift in microbiota composition compared with healthy controls, and gut microbial signature could be used to predict early-stage lung cancer (Zheng et al., 2020). Furthermore, metabolites produced by microbes in the host may play a role in the development of lung cancer. *Granulicatella adiacens* (*G. adiacens*) from sputum contributed to the development of lung cancer, and functional analysis revealed that *G. adiacens* was engaged in polyamine metabolism (Cameron et al., 2017). It is known that polyamines are associated with various diseases, including lung cancer (Nowotarski et al., 2013). A study discovered that the abundance of *Cyanobacteria* in lung tissue was significantly increased in lung adenocarcinoma, and functional analysis suggested that the *Cyanobacteria* toxin could be associated with increases in procyclic acidic repetitive protein 1, which could enhance inflammatory reaction and promote cancer progression (Apopa et al., 2018). The metabolomic profiling of gut microbiota from 11 NSCLC patients demonstrated that 2-pentanone and tridecane were associated with disease progression, and SCFA, lysine and nicotinic acid were associated with long-term treatment effects (Botticelli et al., 2020).

Thus, we launched the study: 1) that uses deep metagenomic profiling of gut microbiomes to discover related microbial genes on baseline fecal samples from LA-NSCLC patients and healthy first-degree relatives; 2) to evaluate the association of baseline gut microbiome signature and disease progression to provide information for clinical practice.

## Materials and Methods

### Study Participants and Design

The metagenome-wide association studies were performed on baseline fecal samples from 18 LA-NSCLC patients before CCRT and 13 controls from healthy first-degree relatives. Patients were selected from a prospective clinical trial (NCT02573506) which evaluated the efficacy of hypofractionated CCRT in LANSCLC. Eligible patients had: 1) confirmed diagnosis of LA-NSCLC and treated with definitive CCRT; 2) age between 18 and 85 years; 3) Karnofsky performance score ≥80; and 4) no gastrointestinal tract disorders or intake of antibiotics/corticosteroids/probiotics within last 8 weeks. Patients were excluded if they had: 1) previous anti-cancer therapy, including chemotherapy, radiotherapy, surgery, or immunotherapy (Khan et al., 2021); intellectual disability or refuse to participate in the study; or 3) history of gastrointestinal surgery, such as small bowel resection. The study was conducted in accordance with the Declaration of Helsinki, and approved by the institutional Ethics Committee of Guangdong Association Study of Thoracic Oncology (GASTO1011 approved on the August 26, 2015, GASTO1017 approved on January 31, 2016.). Informed consents were obtained from all subjects involved in this study. All procedures were conducted in accordance with relevant guidelines.

Among the 18 LA-NSCLC patients, six patients were defined as the long disease progression-free survival (PFS) group (PFS≥11 months) while another 12 were in the short PFS group (PFS<11 months). PFS was calculated as the time from the start of radiotherapy to the date of disease progression or death.

### Sample Collection and DNA Preparation

All stool samples were collected in Sun Yat-sen University Cancer Center, kept in sealed containers with some air at a temperature below 8 °C for transit, then processed within 24 hours after collection. Sample protector for DNA (Takara, 9750) was added in all processed samples and stored in a -80°C freezer. Total DNA was extracted from thawed fecal samples using the QIAamp PowerFecal DNA Kit (Qiagen, Hilden, Germany) following the manufacturer’s instructions. The concentration of genomic DNA in each sample was quantified using a NanoDrop 2000 spectrophotometer (Thermo Scientific). All qualified samples were stored at -20°C for further analysis.

### Metagenomic Sequencing and Data Quality Control

The extracted microbial DNA was processed to construct metagenome shotgun sequencing libraries which were produced using a TruSeq DNA sample preparation kit (Illumina) according to the manufacturer’s instructions. Ten Gb of 150 bp paired-end reads per sample were produced using an Illumina HiSeq platform to assess the proportion of microbial sequences. Sequence quality control and filtering were conducted by fastp (https://github.com/OpenGene/fastp) (Chen et al., 2018). Processing steps, including quality control checks, filtering, and metagenome assembly were performed using MEGAHIT (https://github.com/voutcn/megahit) (Li et al., 2015) and Newbler (version 2.6). Contig binning was executed using CD-HIT (http://www.bioinformatics.org/cd-hit/) (Fu et al., 2012), and genes were predicted using MetaGene (http://metagene.cb.k.u-tokyo.ac.jp/) (Zhu et al., 2010). SOAPaligner software (http://soap.genomics.org.cn/) (Li et al., 2009) was applied to compare the high-quality reads of each sample with the non-redundant gene set (default parameter: 95% identity).

### Identification of Microbial Species, Functional Genes

DIAMOND software (https://github.com/bbuchfink/diamond) (Buchfink et al., 2015) was used to blast the unigenes to the sequences of Bacteria (Parameter: blastp; E-value ≤ 1e-5) which were all extracted from non-redundant protein amino acid sequence database, including SwissProt, Protein Information Resource, Protein Research Foundation, Protein Data Bank, non-redundant protein data, and protein data translated from coding sequence features from GenBank and RefSeq. Species annotation results were obtained through the taxonomic information database corresponding to the NR library, and then use of the sum of the gene abundances corresponding to the species to calculate the abundance of the species. The abundance of species in each sample at each taxonomic level (kingdom, phylum, class, order, family, genus, species) were obtained based on the lowest common ancestor annotation results and then gene abundance table corresponding to each taxonomic level was constructed.

We annotated functional gene clusters and compared them with those (Parameter: blastp; E-value ≤1e-5) in the Kyoto Encyclopedia of Genes and Genomes (KEGG, http://www.genome.jp/kegg/) (Kanehisa et al., 2016). Then we calculated the abundance of the functional category based on the sum of the gene abundances corresponding to KEGG Orthology (KO), Pathway, Module and Antibiotic Resistance Genes Database (ARDB, http://ardb.cbcb.umd.edu/) through DIAMOND (https://github.com/bbuchfink/diamond). The annotation information of antibiotic resistance function corresponding to gene was obtained, and then the abundance of antibiotic resistance function was calculated using the sum of gene abundance corresponding to antibiotic resistance function.

### Statistical Analysis

SPSS (version 26.0), GraphPad Prism (version 7.00), and R software (version 3.5.2) were used for statistical analysis. Parameter differences among three groups, including Firmicutes/Bacteroidetes (F/B), alpha diversity (Shannon index), and number of antibiotic resistance genes, were tested using one-way ANOVA for multiple comparisons. Comparisons between long-PFS and short-PFS groups were performed with Wilcoxon rank sum test or LEfSe (http://huttenhower.sph.harvard.edu/galaxy) for quantitative or categorical variables, respectively. P-values were corrected for multiple comparisons with Benjamini-Hochberg method for the false discovery rate.

## Results

### Clinical Characteristics of Participants

The clinical characteristics of 18 LA-NSCLC patients and 13 first-degree relatives are detailed in **Table 1**. Among the 18 LA-NSCLC patients, six patients were long-PFS group (PFS≥11 months) while another 12 were in the short-PFS group (PFS<11 months). The baseline clinical characteristics of these three groups were comparable.

**Table 1:** The baseline clinical characteristics of study populations.

Characteristics	Short-PFS group (n=12)	Long-PFS group (n=6)	Healthy individuals (n=13)	P value
Age, y				
Median (Range)	59 (35–69)	55 (50-63)	53 (36-66)	0.093
Sex, n (%)				
Male	10 (83.3)	4 (66.7)	7 (53.8)	0.288
Female	2 (16.7)	2 (33.3)	6 (46.2)	
Body mass index (kg/m2)				
Mean (SD)	24.48 (2.81)	23.77 (3.20)	23.52 (3.10)	0.721
Smoking history, n (%)				
Smoker	8 (66.7)	4 (66.7)	7 (53.8)	0.770
Non-smoker	4 (33.3)	2 (33.3)	6 (46.2)	
Histology, n (%)				
Squamous cell carcinoma	7 (58.3)	5 (83.3)	/	0.600
Adenocarcinoma	5 (41.7)	1 (16.7)	/	
Disease stage, n (%)				
IIIA	1 (8.3)	1 (16.7)	/	0.957
IIIB	8 (66.7)	3 (50.0)	/	
IIIC	3 (25.0)	2 (33.3)	/	
Chronic lung comorbidity				
Yes	5 (41.7)	1 (16.7)	/	0.600
No	7 (58.3)	5 (83.3)	/	

### Taxonomic Analysis of Metagenomic Sequence Data

**Figure 1:**
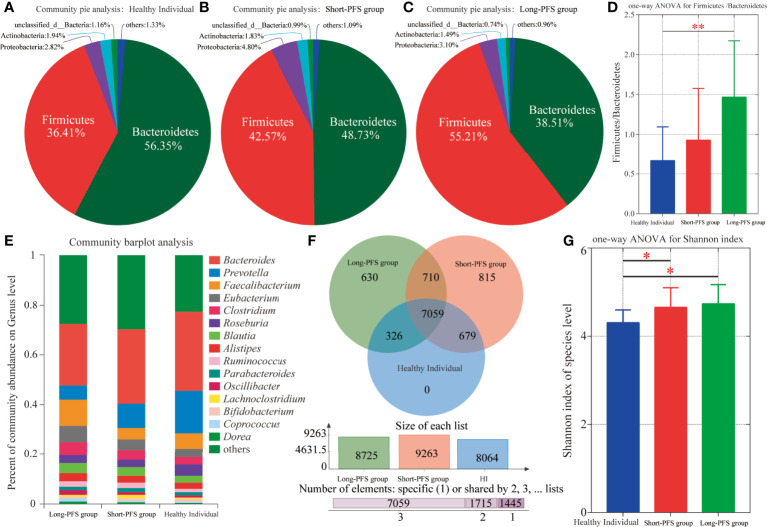
Intestinal microbiota composition among long-PFS group, short-PFS group and healthy individuals (HI). **(A-C)** The community pie analysis at phylum level for HI, short-PFS group and long-PFS group, respectively. **(D)** The one-way ANOVA of the *Firmicutes/Bacteroidetes* ratio among three groups. **(E)** Stacked bar plots depicting genus-level differences in gut microbiota composition. **(F)** The venn diagram analysis among three groups at species level. **(G)** Comparison of microbial alpha diversity (shannon index) among three groups. *P < 0.05, **P < 0.01.

**Figure 2:**
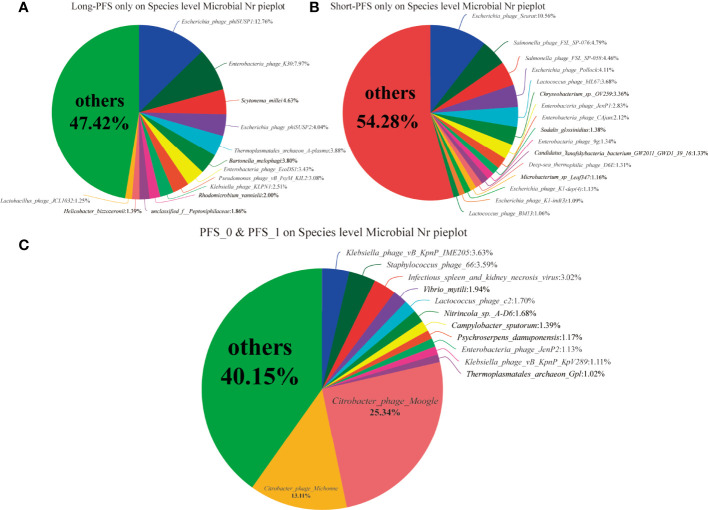
**(A–C)** The community pie analysis at speices level only on long-PFS group, only on short-PFS group and both long-PFS group and short-PFS group, respectively.

In all the study population, the proportions of domains at species level were Bacteria 83.87%, Viruses 6.5%, Eukaryota 6.46%, Archaea 3.25%, and unclassified 0.23%. The relative abundance of Bacteria accounted for 99.45%. The abundance of Bacteroidetes and Firmicutes were different in long-PFS, short-PFS, and healthy individual (38.51% vs. 48.73% vs. 56.35%; 55.21% vs. 42.57% vs. 36.41%, respectively) (**Figures 1A–C**). The Firmicutes/Bacteroidetes (F/B) value of long-PFS group was higher than short-PFS (p=0.073) and healthy individual (p=0.009) (**Figure 1D**). The composition and structure of gut microbiomes in the three groups were also different. The top 15 genera at the genus level were *Bacteroides*, *Prevotella*, *faecalibacterium*, *Eubacterium*, *Clostridium*, *Roseburia*, *Blautia*, *Alistipes*, *Ruminococcus*, *Parabacteroides*, *Oscillibacter*, *Lachnoclostridium*, *Bifidobacterium*, *Coprocccus*, and *Dorea* (**Figure 1E**). There were 7059 species in the three groups in common at species level, and 630 and 815 species were unique to long-PFS and short-PFS group, respectively (**Figure 1F**). Further analysis of the unique species demonstrated that various bacteriophages could be found on these two groups (**Figures 2A–C**), resulting in a significant higher alpha diversity (shannon index) in short-PFS group (P=0.029) and long-PFS group (P=0.021) compared with healthy control group (**Figure 1G**).

**Figure 3:**
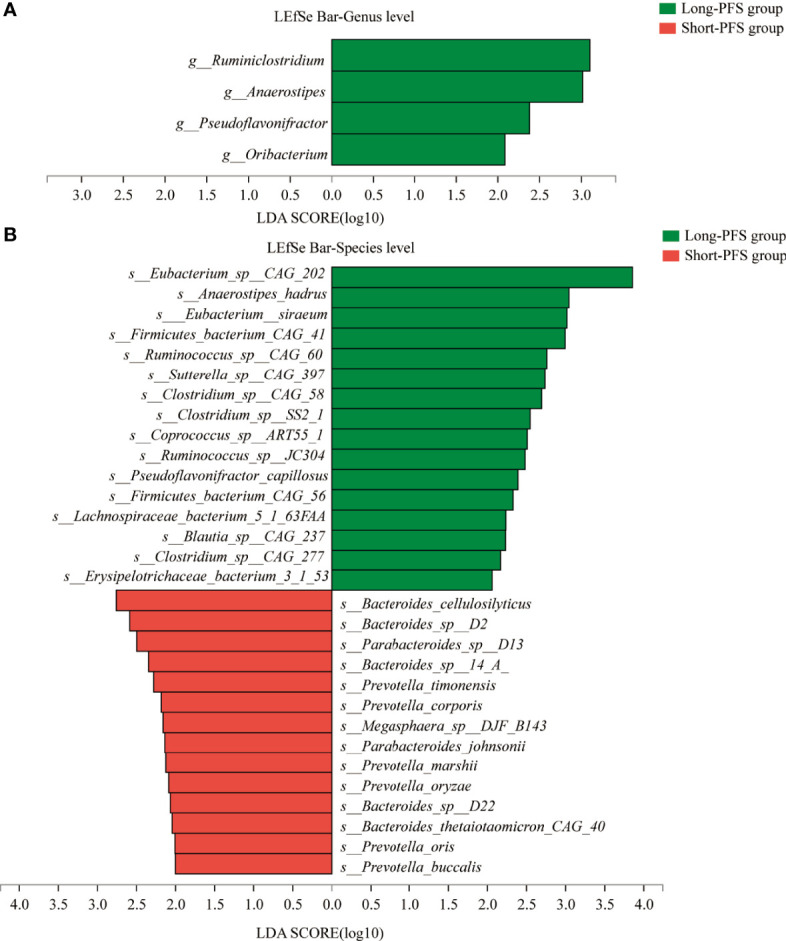
The LEfSe analysis between short-PFS group and long-PFS group at genus level **(A)** and species level **(B)**.

To explore the differences of gut microbiota between short-PFS and long-PFS group, LEfSe analysis was performed with an LDA score of 2.0. At genus level, there are four specific genera from the long-PFS group, including *Ruminiclostridium* (LDA=3.102), *Anaerostipes* (LDA=3.015), *Pseudoflavonifractor* (LDA=2.379), and *Oribacterium* (LDA=2.084) (**Figure 3A**). At species level, there are 30 specific species between two groups, of which 14 species in short-PFS group and 16 species in long-PFS group. The highest LDA score was for *Eubacterium_sp_CAG_202* (LDA=3.857) in long-PFS group, and *Bacteroides_cellulosilyticus* (LDA=2.760) in short-PFS group (**Figure 3B**). Meanwhile, in Eukaryota, Archaea, and Viruses, the main features were in long-PFS group and the number of Fungi features were the most, 109 distinguishing characteristics (**Figures S1 A–C**).

### Functional Profile of the Gut Microbiome

**Figure 4:**
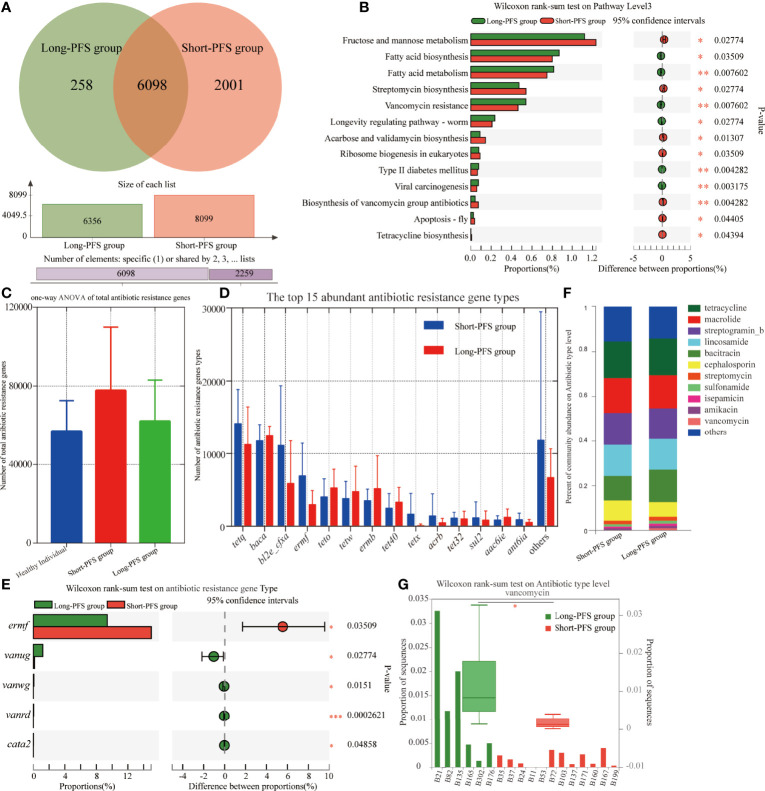
Differences in functional profile of gut microbiome between patients in long-PFS and short-PFS groups. **(A)**The venn diagram analysis between short-PFS group and long-PFS group in KEGG ORTHOLOGY (KO). **(B)** Significant difference was observed by Wilcoxon rank-sum test at KEGG level 3 between short-PFS and long-PFS groups. **(C)** The one-way ANOVA of total antibiotic resistance genes. **(D)** The histogram shows the top 15 abundant antibiotic resistance gene types in long-PFS and short-PFS groups. **(E)** Significant difference was observed by Wilcoxon rank-sum test at antibiotic resistance gene types. **(F)** Comparison of the top 11 most abundant antibiotic resistance genes between two groups. **(G)** The Wilcoxon rank-sum test analysis of vancomycin resistance gene types between two groups. *P < 0.05, **P < 0.01, and ***P < 0.001.

The KEGG analysis of the metagenomic data revealed 8357 KO, with 6098 presented in both long-PFS and short-PFS groups. Short-PFS group had more unique KO compared with long-PFS group (2001 vs. 258) (**Figure 4A**). The KEGG pathways overrepresented in short-PFS group included fructose and mannose metabolism (p=0.028), streptomycin biosynthesis (p=0.028), acarbose and validamycin biosynthesis (p=0.013), ribosome biogenesis in eukaryotes (p=0.035), biosynthesis of vancomycin group antibiotics (p=0.004), apoptosis-fly (p=0.044), and tetracycline biosynthesis (p=0.044); while those overrepresented in long-PFS group included fatty acid biosynthesis (p=0.035), fatty acid metabolism (p=0.008), vancomycin resistance (p=0.008), longevity regulating pathway-worm (p=0.028), type II diabetes mellitus (p=0.004), and viral carcinogenesis (p=0.003) (**Figure 4B**).

### Abundance of Antibiotic Resistance Genes

The resistome was characterized by identifying antibiotic resistance genes annotated in ARDB (Antibiotic Resistance Genes Database) from the metagenomes of each sample using the Short, Better Representative Extract Data set. The short-PFS group had a trend with more antibiotic resistance genes than healthy control (p=0.070) and long-PFS groups (p=0.218) (**Figure 4C**). The top 15 most abundant antibiotic resistance gene types varied between the short-PFS group and the long-PFS group, and the most abundant type was *tetQ* in short-PFS group and *Baca* in long-PFS group (**Figure 4D**). Other differentially enriched antibiotic resistance genes included *Ermf* enriched in short-PFS group (p=0.035), *Vanug*, *Vanwg*, *Vanrd* and *Cata2* enriched in long-PFS group (p=0.028; p=0.015; p<0.001; p=0.049, **Figure 4E**). The corresponding main types of antibiotics were tetracycline, macrolide, aminoglycosides (streptogramin_b, streptomycin, isepamicin, amikacin), lincosamide, polypeptide (bacitracin, vancomycin), β-lactams (cephalosporin), and sulfonamide (**Figure 4F**). The vancomycin resistance sequences were significantly enriched in the long-PFS group compared to the short-PFS group (p=0.006) (**Figure 4G**). Further analysis of functional metabolic pathway related to the KEGG pathway biosynthesis of vancomycin group antibiotics found that the dTDP-4-dehydro-6-deoxy-alpha-D-glucopyranose and 2,3-dehydratase were abundant in long-PFS group (p=0.35), while dTDP-glucose4,6-dehydratase was abundant in short-PFS group (p=0.35) (**Figure S2**).

**Figure 5:**
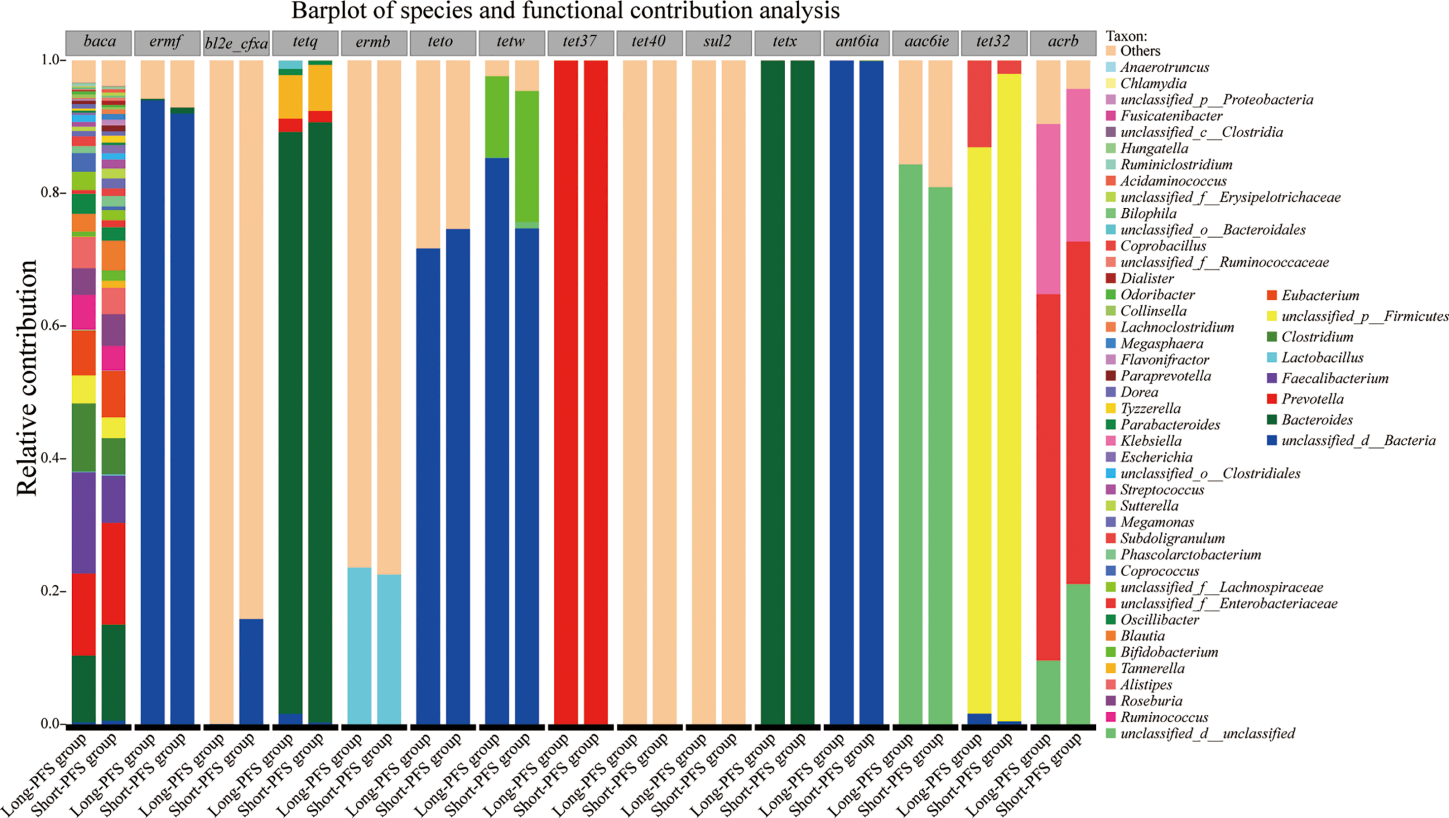
Barplot of species and functional contribution analysis demonstrating the top 50 genus contributing to the top 15 antibiotic resistance gene types.

To discover which bacterial genera contributed to the ARDB reservoir, we performed a species and functional contribution analysis of ARDB type and the top 50 bacterial genera. The main carrier for antibiotic resistance genes was *Bacteroides*, followed by *Prevotella* and *Faecalibacterium*. *Baca* had a broad spectrum of carriers, among which *Prevotella* accounted for the largest proportion, with 12.34% in long-PFS and 15.31% in short-PFS group. *TetQ* and *TetX* mainly came from *Bacteroides*, while *Tet37* all came from *Prevotella* (**Figure 5**).

## Discussion

Concurrent chemoradiotherapy (CCRT) is the standard treatment for locally advanced non-small cell lung cancer (LA-NSCLC). The outcome of CCRT varies among patients due to many clinical and biological factors. Recent studies indicate that gut microbiota play a vital role in modulating host immunological responses to multiple therapies. Therefore, we aimed to find the association between baseline microbiome signatures and treatment outcome in LA-NSCLC treated with CCRT. We had launched a prospective clinical trial which assessed the efficacy of hypofractionated CCRT in LANSCLC, with a median PFS of 11 months (Qiu et al., 2021). The current study was an exploratory analysis of the clinical trial evaluating the association of baseline gut microbiome and treatment outcome. In the present study, baseline gut microbiome has been investigated in LA-NSCLC patients treated with CCRT and first-degree relatives, aiming to provide information for clinical practice.

Recent studies have demonstrated that gut microbiome is associated with NSCLC progression. Patients with higher gut microbiota alpha diversity (Zhang C. et al., 2021), and those enriched in some microbes, such as *Phascolarctobacterium* (Zhang F. et al., 2021), *Ruminococcaceae UCG 13*, and *Agathobacter (* Hakozaki et al., 2020), were found to have better PFS. In the current study, we found that the long-PFS group had the highest F/B value and significantly higher diversity in Fungi, Archaea, and Viruses among three groups. F/B value is frequently reported in various pathophysiological process including metabolism, nutrition, and cancer development. A study focusing on the role of the microbiome in obesity and diabetes in humans and rodents revealed significant differences of gut microbiota between lean and obese subjects, most notably an increased F/B ratio in obesity subjects (Ley et al., 2006). Analysis of non-alcoholic fatty liver disease patients also showed an elevated F/B ratio compared with healthy control (Yu et al., 2021). In addition, higher risk of breast cancer recurrence was reported to be associated with lower F/B ratio (Okubo et al., 2020). Firmicutes were less frequent in colorectal cancer than in the corresponding normal mucosa (Gao et al., 2017), and a lower F/B ratio may be related with colorectal cancer progression and recurrence. In our study, we found that the F/B ratio was a favorable indicator for PFS, which was consistent with previous reports. The balanced biodiversity might contribute to long-term survival in patients with LA-NSCLC treated with CCRT. The gut microorganisms, as is widely known, are composed of bacteria, viruses, fungi, archaea, and microscopic eukaryotes, developing intricate symbiotic and mutualistic relationships. To sustain various host-beneficial functions, the host and gut flora interactions must be in a state of equilibrium. Variations in the taxonomic and functional composition of gut microbes have been related to human disorders and the preservation of a healthy state. There is mounting evidence that non-bacteria gut microorganisms play a role in cancer pathogenesis as well. Recent research has indicated that bacteria, viruses, and/or fungi were widespread in malignancies, played critical actors in cancer immunotherapy, and could be manipulated to reduce the incidence of metastases (Coker et al., 2019; Hakozaki et al., 2020; Zhang L. et al., 2021; He et al., 2021). Our study also found various bacteriophages in NSCLC patients, which indicated that other non-bacteria gut microorganisms like virus may be important in gut homeostasis in disease states.

Metabolites produced by gut microbiota could be absorbed and enter the circulation, and then reach distal tissues from intestines and influence cancer development (Fujisaka et al., 2018; Xavier et al., 2020). For example, gut microbes can convert primary bile acids to secondary structures, which could regulate immune response to hepatocellular carcinoma cells (Ma et al., 2018). Some gut microbes, which could metabolize estrogen, potentially changing the risk of postmenopausal breast cancer, regardless of the long distance between breast and gut (Kwa et al., 2016). In our study, Fatty acid metabolism and vancomycin resistance were overexpressed in long-PFS group. Accordingly, *g_Anaerostipes*, a SCFA-producing microbe, was enriched in long-PFS group. SCFAs, including butyrate, propionate, and acetate, are important metabolites in fatty acid metabolism. SCFAs could exert various beneficial effects, such as promoting metabolism, maintaining immune homeostasis, and providing protection against cancers (Mirzaei et al., 2021). A study has found that butyrate could inhibit proliferation of lung cancer by modulating p21 expression, and propionate could inhibit cell growth by inducing cell apoptosis and cell cycle arrest (Kim et al., 2019). In addition, a recent study reported that SCFAs tended to be lower in cachectic cancer patients, which indicated that SCFAs may be crucial for clinical nutrition in cancer patients (Ubachs et al., 2021). It is known that vancomycin mainly acts on gram-positive bacteria, including some butyrate-producing bacteria, which may lead to SCFA concentrations decreased (Uribe-Herranz et al., 2020). Therefore, we inferred that those bacteria with vancomycin resistance genes in long-PFS group might survive under vancomycin selection pressure and help to maintain SCFA concentrations in host, which needs to be validated in future studies. In addition, we also found that fructose and mannose metabolism was overrepresented in short-PFS group. D-sorbitol and D-mannose metabolites were important in fructose and mannose metabolism. However, few studies have reported the function of those metabolites in cancers. Further metabolomics analysis is needed to figure out the abundance of those metabolites between different prognostic groups and discover the relationship between carbohydrate metabolism pathways and prognosis.

Our study demonstrated that multiple antibiotics biosynthesis (streptomycin, validamycin, vancomycin, and tetracycline) pathway was overexpressed in short-PFS group. Many patients with LANSCLC were diagnosed with pre-existing lung diseases, such as chronic obstructive pulmonary disease. These patients had more exposure to antibiotics, and those gut bacteria which have corresponding antibiotic biosynthesis genes could be selected and survived. In the current study, five patients (41.7%) in short-PFS group and one patient (16.7%) in long-PFS group had chronic lung comorbidity (**Table 1**). More exposure to antibiotics due to lung comorbidity might contribute to the overexpression of multiple antibiotics biosynthesis in short-PFS group. Antibiotics biosynthesis of gut microbiota might explain why the short-PFS group had more antibiotic resistance genes than other groups. Antibiotics synthesized and released locally may induce dysbiosis and alter the diversity of gut microbiota. Those bacteria which have corresponding antibiotic resistance gene could be selected and survive. Several studies have indicated that antibiotic use would have a negative impact on the gut microbiota, including decreased diversity, altered metabolic activity, and selected antibiotic-resistant organisms, which may lead to unfavorable treatment response (Elkrief et al., 2019; Gao et al., 2021; Oh et al., 2021). A study found that using antibiotics before immunotherapy in LA-NSCLC patients would lead to lower alpha diversity at baseline and underrepresentation of *Ruminococcaceae UCG 13* and *Agathobacter*, which were enriched in patients with favorable anti-tumor response and survival outcomes (Hakozaki et al., 2020).

Antibiotics, diet, and excessive cleanliness may alter the composition of the microbiome in our gut (Xavier et al., 2020). Antibiotics could alter the gut microbiota and have an influence on their metabolites, which can affect normal tissue cells which interact with malignancies in distant regions (Schulfer et al., 2019). Trimethylamine N-oxide and betaine, two metabolites regulated indirectly by antibiotic usage, have an effect on macrophages, modifying their phenotype associated with atherosclerosis (Wang et al., 2011). In that case, the antibiotic therapy altered the microbiota, which in turn influenced the amounts of circulating metabolites in a way that compromised host health. Previous studies have indicated a positive correlation between antibiotic usage and the degree of resistance (Laxminarayan et al., 2016). Antibiotic treatment damaged the balance between host and its gut microbes, leading to the generation of antibiotic-resistant strains (Schwartz et al., 2020). Antibiotic abuse and the related resistance concerns are quite significant in China (Heddini et al., 2009). When compared to other nations, China has the fastest rate of resistance generation as well as the largest amount and diversity of antibiotic resistance genes (ARG) (Zhang et al., 2006). It could be deduced that, due to relatively lax antibiotic supervision in China in recent years, antibiotics have been used and accumulated in significant quantities across the whole lifespan. This could be the reason why the elderly in China possessed the highest amount of ARG. Nevertheless, there are still many issues that need to be investigated and identified, like how these resistance genes are gained and distributed.

Gut microbe acts as a reservoir for spreading antibiotics resistance genes from commensals to pathogens, which is termed gut resistome (Montassier et al., 2021). Our study showed that different antibiotic resistance genes were enriched in short-PFS and long-PFS groups. Bacteria enriched in *Ermf* genes had resistance of macrolide, lincosamide, and streptogramin, while microbiota enriched in *Vanug*, *Vanwg*, and *Vanrd* had vancomycin resistance. The metagenomic analysis of antibiotic resistome in a large-scale healthy human demonstrated that Chinese population harbored the most abundant ARGs, and *Ermf* could be representative ARGs of the Chinese population (Qiu et al., 2020). Except for antibiotics abuse, ARGs could be transferred horizontally through bacteria from food and animals, which means ARGs could be obtained through various ways such as the transmission of food chain (Kumar et al., 2020). The relationship between gut resistome and cancer development needs to be further investigated in the future.

The current study has several limitations. Firstly, the samples are collected from 18 patients and 12 first-degree relatives, which may not be sufficiently representative. Integrating data from various subgroups may assist in obtaining new insights. Secondly, the sample distribution is not homogeneous, and the antibiotic medication history of each individual was hard to clarify. It might result in biased identification of specific ARG markers. To reach a broader and fairer conclusion, various kinds of cancers should be investigated, as well as analyses of their ARG markers between distinct cohorts. Thirdly, further investigation and validation in large cohorts needs to be conducted in the future.

## Conclusions

The baseline gut microbiome composition and functionality might be associated with PFS in LA-NSCLC treated with CCRT. The higher baseline microbiome diversity related to long PFS and the outcome of CCRT might be modulated through bacterial metabolic pathways. The antibiotic resistance genes might play a role in disease progression and provide potential information on the relationship between the use of antibiotics and treatment efficacy of CCRT in LA-NSCLC. Further research is needed to confirm these results.

## Data Availability Statement

The datasets presented in this study can be found in online repositories. The names of the repository/repositories and accession number(s) can be found below: https://www.ncbi.nlm.nih.gov/bioproject/PRJNA746114.

## Ethics Statement

The studies involving human participants were reviewed and approved by the institutional Ethics Committee of Guangdong Association Study of Thoracic Oncology. The patients/participants provided their written informed consent to participate in this study.

## Author Contributions

YX: Data curation, Conceptualization, Methodology, Formal analysis, Investigation, Writing—original draft, Writing–review and editing, Visualization. FL: Investigation, Formal analysis, Writing—original draft, Writing—review and editing. BQ: Conceptualization, Methodology, Formal analysis, Writing—review and editing. YL: Conceptualization, Methodology, Investigation. XX: Conceptualization, Methodology, Formal analysis, Investigation, Writing—review and editing. JG: Methodology, Investigation. LW: Methodology, Investigation. TL: Methodology, Investigation. DW: Methodology, Investigation. JW: Conceptualization, Methodology, Investigation, Writing—review and editing. MC: Methodology, Investigation. LX: Methodology, Investigation. YD: Conceptualization, Methodology, Investigation, Writing–review and editing. JZ: Conceptualization, Methodology, Resources, Writing—review and editing, Funding acquisition. QW: Conceptualization, Methodology, Resources, Writing—review and editing, Funding acquisition, Supervision, Validation. HL: Conceptualization, Methodology, Formal analysis, Writing—review and editing, Supervision, Validation. All authors have read and agreed to the published version of the manuscript.

## Funding

This study was supported by the National Natural Science Foundation of China (Grant Number 82073328), National Key R&D Program of China (Grant Number 2018YFC0116800), the Guangdong Province Academy of Sciences Special Project for Capacity Building of Innovation Driven Development (Grant Number 2020GDASYL-20200301002), and Key Laboratory of Guangdong Province (Grant Number 2020B121201009). GDAS' Special Project of Science and Technology Development (2019GDASYL-0201001). The GDAS Project of Science and Technology Development (2019GDASYL-0103008).

## Conflict of Interest

The authors declare that the research was conducted in the absence of any commercial or financial relationships that could be construed as a potential conflict of interest.

## Publisher’s Note

All claims expressed in this article are solely those of the authors and do not necessarily represent those of their affiliated organizations, or those of the publisher, the editors and the reviewers. Any product that may be evaluated in this article, or claim that may be made by its manufacturer, is not guaranteed or endorsed by the publisher.
