# Supplementary material for: Analysis of Gut Microbiota Signature and Microbe-Disease Progression Associations in Locally Advanced Non-Small Cell Lung Cancer Patients Treated With Concurrent Chemoradiotherapy
Source: Front Cell Infect Microbiol. 2022 Jun 1;12:892401. doi: 10.3389/fcimb.2022.892401 (PMC9200620; doi:10.3389/fcimb.2022.892401)
Supplement: Supplementary file 1 [file DataSheet_1.docx]

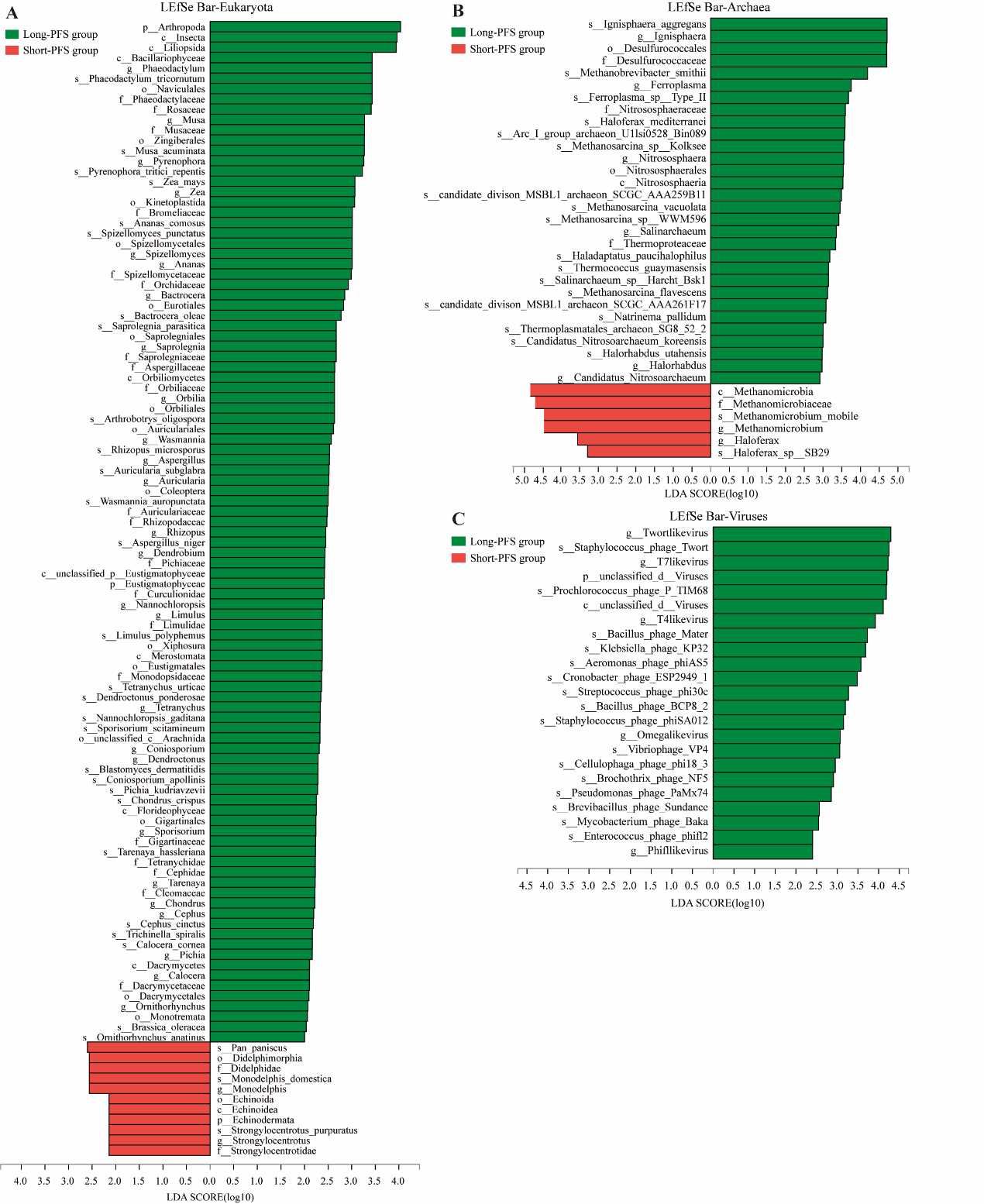


**Figure S1.** The LEfSe analysis between short-PFS group and long-PFS group in Eukaryota (A), Archaea (B) and Viruses (C).


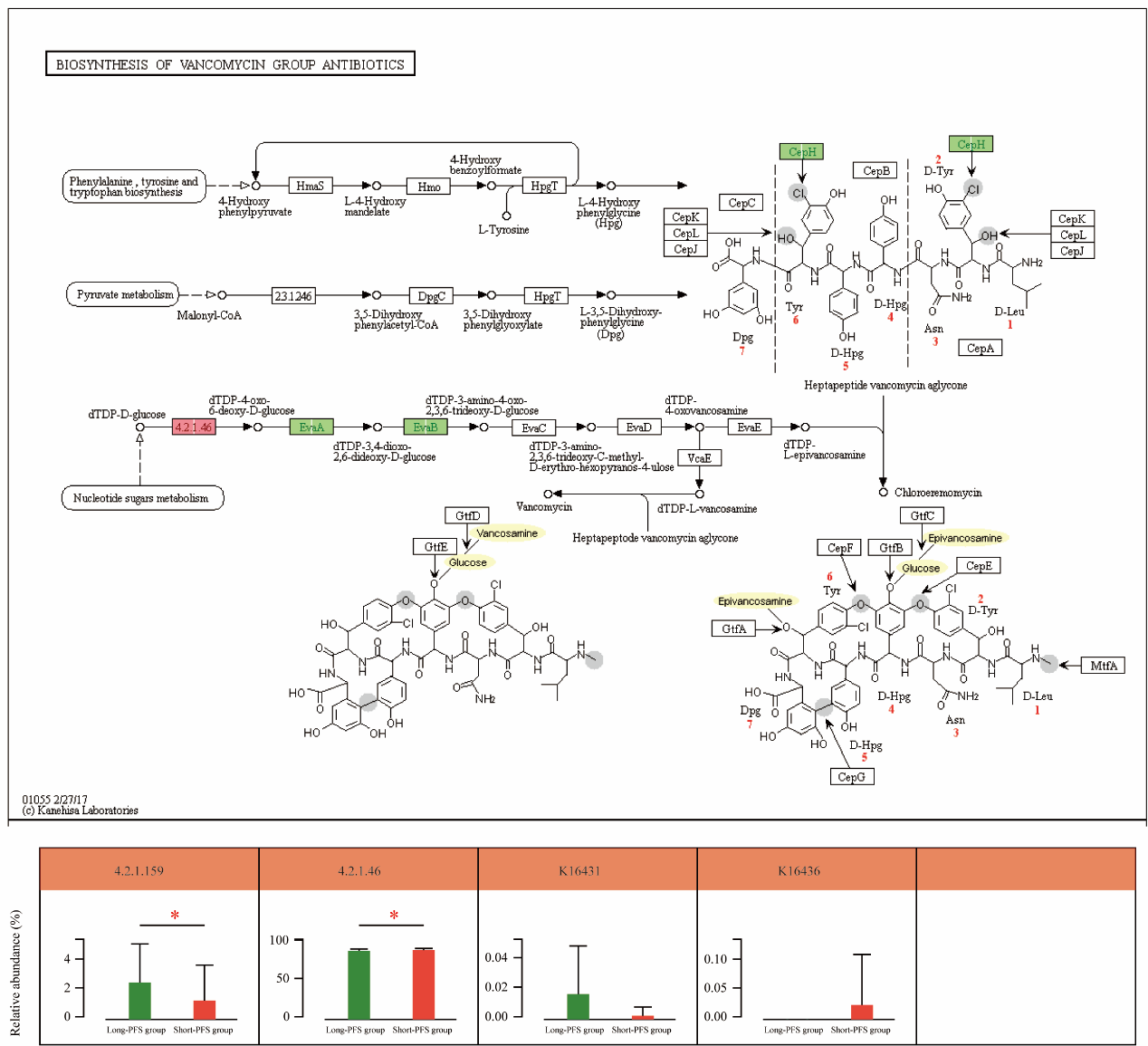


**Figure S2.** The Wilcoxon rank-sum test analysis of metabolic pathways in biosynthesis of vancomycin group antibiotics between short-PFS group and long-PFS group.
